# Perceptions of gender equality, work environment, support and social issues for women doctors at a university hospital in Riyadh, Kingdom of Saudi Arabia

**DOI:** 10.1371/journal.pone.0186896

**Published:** 2017-10-26

**Authors:** Shehla Baqi, Amal Albalbeesi, Sundus Iftikhar, Naila Baig-Ansari, Mohammad Alanazi, Awadh Alanazi

**Affiliations:** 1 Department of Medicine, College of Medicine, King Khalid University Hospital, King Saud University, Riyadh, Kingdom of Saudi Arabia; 2 Department of Dermatology, College of Medicine, King Khalid University Hospital, King Saud University, Riyadh, Kingdom of Saudi Arabia; 3 Indus Hospital Research Center, Indus Hospital, Korangi Crossing, Karachi, Pakistan; 4 Department of Physical Medicine and Rehabilitation, College of Medicine, King Khalid University Hospital, King Saud University, Riyadh, Kingdom of Saudi Arabia; National Academy of Medical Sciences, NEPAL

## Abstract

The Kingdom of Saudi Arabia (KSA) is an Islamic monarchy and was established in 1932. Saudi women first entered the medical field in 1975 and the country has since seen a steady increase in women pursuing medicine. However, there is limited data on gender related issues for women doctors practicing in Saudi Arabia. Therefore, our study objective was to assess the perception amongst peers regarding gender equality and social issues faced by women doctors in Saudi Arabia. An online anonymous cross-sectional survey was administered in English to doctors at King Khalid Hospital, affiliated to King Saud University, in Riyadh, between April and May of 2016. Of 1015 doctors, 304 (30%) participated, of which 129 (42.4%) were females and 231 (76%) were Saudi nationals. The average age was 32.4 years (±SD: 8.7). The majority opined that there was no gender discrimination in salaries (73.7% p-value = 0.4), hospital benefits (62.2% p-value = 0.06) or entry into any field of Medicine/Pediatrics (68.4% p-value = 0.207). However, only a minority believed that there was no gender discrimination for entry into surgery (37.3% p-value = .091). A higher proportion of male doctors agreed that promotion opportunities are equal (66.3% vs 45.7%, p-value = 0.002). However, of 54 consultants, only 18 (33.3%) were women. Over half of the women (52.3%) reported that they never wear the face veil. Only a minority of male and female doctors (12.2%) believed women doctors should wear the veil since they examine male patients. Fewer respondents believed that female doctors face harassment from male doctors (14.5%) whereas 30.7% believed female doctors face harassment from male patients. More females, than males, agreed with the statement that female doctors are as committed to their careers as are males (92.2% vs 67.4%, p-value<0.0001). Of 304 participants, 210 (69.1%) said that they would still choose to become a doctor with approximately equal proportions between males and females (68% vs 70.5%, p-value = 0.79). In conclusion, our survey of male and female doctors at a government university hospital in Saudi Arabia revealed that the majority believed there was gender equality amongst doctors in terms of salaries, benefits, opportunities for promotion and entry into any field of medicine or pediatrics, but not surgery. However, there were significantly fewer women at consultant positions, a deficiency that needs to be addressed.

## Introduction

The Kingdom of Saudi Arabia (KSA), established in 1932, is the largest Arab state. [1] It is an Islamic monarchy with a population of over 30 million, two-thirds of which are Saudi nationals. [2, 3] The World Economic Forum's 2016 Global Gender Gap Report has ranked Saudi Arabia 141 out of 144 countries for gender parity. [4] In 2015, Saudi women constituted only 13% of the native work force. [5] However, the number of employed Saudi women with professional careers is increasing, made possible by free education and rapid expansion of institutes of higher education. [6–9] These include King Saud University, the country's first University and medical school and co-incidentally our study site, the Princess Nourah Bint Abdulrahman University, the world's largest women-only university, and King Abdullah University of Science and Technology with the first co-educational campus. [10, 11] Nearly 60% of university graduates in Saudi Arabia are women, and the female youth literacy rate (ages 15–24) is 99.3%. [11–13] By 1960, 5% of medical students were women in the United States [14] which increased to nearly 47% in 2014. However, in Saudi Arabia, women did not enter the medical field until 1975, but there has been a steady increase in the numbers of female graduates over the years. [15, 16] In 2016, of 18728 medical graduates, 7590 (40.5%) were women in Saudi Arabia. [17] A study of Saudi secondary school female graduates in 1998 regarding a career in medicine reported concerns about mixing with males (16.7%), and balancing career with family life (12.9%). [18] Today, about 38% of doctors in Saudi Arabia are nationals, with women constituting one-third of all Saudi doctors. [16, 19] Saudi Arabia is one of the wealthiest countries in the world and, in many respects, as modern as any other in terms of public health services, transportation and infrastructure. Teaching hospitals in Saudi Arabia are well funded and doctors follow a western (mostly North American) style of practice, documentation, undergraduate and post-graduate training, performance evaluations and examinations, all of which are conducted in the English language. These similarities allow comparison of this Saudi study of gender equality in the medical field with similar studies conducted in western countries over the past two decades. Western data report increasing parity in numbers, but not in terms of sexual harassment, gender bias, salary and leadership positions. [14, 20–29] There are few studies of gender issues for female doctors in Saudi Arabia, with many more anecdotal subjective perceptions. [30, 31] We hope that our findings will be informative for Saudi girls who are contemplating the medical profession but have concerns, along with their families, regarding its suitability and the prospect of working in a mixed environment. Furthermore, our study provides an opportunity for the Saudi government to address deficiencies identified, given that at present there is sparse data to guide them. Since this influx of women in the medical field in Saudi Arabia is a relatively recent development, our study objective was to survey the opinion of doctors regarding gender equality and social issues faced by women doctors and to further assess differences in perception between male and female doctors.

## Methods and materials

This cross-sectional study was carried out at the King Khalid University Hospital (KKUH), Riyadh, Saudi Arabia. KKUH is a government teaching hospital that opened in 1982 as a dedicated university hospital. It is affiliated to King Saud University and is an 800-bed facility with all general and subspecialty medical services. It consists of mostly government doctors as well as some private contractual doctors. Study participants were doctors employed full-time, irrespective of nationality, in the clinical fields of Medicine, Surgery, Pediatrics, Obstetrics-Gynecology, Primary Care, Family and Community Medicine, Emergency Medicine, Critical Care, Psychiatry and Dermatology at King Khalid Hospital.

Study tools and minimum sample size required were assessed based on a pilot survey conducted in December 2015. The purpose of the pilot was primarily to evaluate the willingness of doctors to participate in a survey that touched upon controversial issues in Saudi Arabia, including governmental policies, religious and social norms. The survey was provided to departmental secretaries in individual envelopes to be distributed to their doctors. Of 800 questionnaires distributed, only 115 responses were received (14.4%). We attributed the poor response to the effort required by busy doctors to manually fill and submit questionnaires. Furthermore, it is conceivable that doctors were not sure regarding confidentiality since sealed envelopes could be opened prior to reaching the Principal Investigator. On this basis, it was decided to create an online survey which would be convenient and would also allow us to retain all questions of a sensitive nature. Moreover, the online survey would additionally reach those fulltime doctors at King Khalid University hospital that may be on leave or on temporary rotation at another facility. We estimated that a total of 1015 doctors met the study criteria and will receive the online link to our study. The sample size was calculated using the pilot data of 115 participants. Open Epi software was used to calculate the sample size based on a 5% desired precision and assuming there are1015 doctors at KKH. Sample size was calculated separately for the various aspects of interest in the study and the highest sample size of 264 was chosen. We added a 20% attrition rate to it, arriving at a required sample size of 310.

The online survey was created using Google Forms in English. The main themes covered by the questionnaire included gender equality, work environment, harassment, support by department and supervisors, and social issues of women doctors. Perceptions of equality and social issues were assessed within these thematic areas using a Likert scale. Respondents were asked whether they agree, disagree or were neutral/uncertain on questions pertaining to salaries, benefits, duty hours, promotion, evaluation, etc. Both male and female respondents were asked whether they agreed, disagreed or were neutral/uncertain on questions pertaining to gender equality in their salaries, benefits, duty hours, and promotion. Furthermore, some of the questions covered in social aspects included if there could be a negative impact on the marriage if a woman doctor earned more than her husband, whether women should also contribute to the household and/or family expenses or if women doctors should observe the veil while examining male patients. In addition, demographical information such as nationality, age, gender and marital status was included.

After permission from departmental chairpersons, an email with the link to the online questionnaires was sent to departmental secretaries for forwarding to all Saudi as well as non-Saudi doctors on their mailing list. The secretaries also sent weekly reminders. The link was available online from April 27^th^ till May 28^th^, 2016.

### Ethical concerns

The study was approved by the Institutional Review Board of King Saud University. The survey was anonymous and all potential identifiers such as department and the country of origin, were excluded.

#### Statistical analysis

Data was transferred from Google forms for analysis into Statistical Package for Social Sciences (SPSS) version 21.0. Mean ± SD was computed for quantitative variables like age, working hours, household hours and sleeping hours. Frequency and percentage were computed for all the qualitative variables. Independent sample T-test/ANOVA was applied as appropriate to assess significant differences in various quantitative variables between male and female doctors, married male and female doctors and doctors who have children. Chi-square test/Fisher-exact test/Likelihood ratio Chi-square test was applied as appropriate to assess significant association among various categorical variables. P-value <0.05 was considered significant.

## Results

### Demographics

Of an estimated 1015 doctors, 304 (30%) participated in the online survey, of which 129 (42.4%) were women and nearly 76% were Saudi nationals. Participants included 141 residents (46.4%); 54 consultants (17.8%) and 43 interns (14.1%) (Table 1). Over half were currently married (55%) and of the ever-married participants, a third had a spouse who was also a doctor. Of 64 participants whose spouse was a doctor, a significantly higher proportion of women doctors were married to doctors than their male counterparts (47.1% vs 31.1%, p-value = 0.003, Table A in S1 File).

**Table 1:** Demographic characteristics of 304 doctors at King Khalid University Hospital, Riyadh.

	Female; 129 (42.4%)	Male; 175 (57.6%)	Total	P-value
Professional Level; n (%)				
Intern	18 (14)	25 (14.3)	43 (14.1)	0.206^†^
Resident	64 (49.6)	77 (44)	141 (46.4)	
Fellow	4 (3.1)	12 (6.9)	16 (5.3)	
Consultant	18 (14)	36 (20.6)	54 (17.8)	
Registrar	7 (5.4)	6 (3.4)	13 (4.3)	
Senior Registrar	8 (6.2)	11 (6.3)	19 (6.3)	
Demonstrator	8 (6.2)	3 (1.7)	11 (3.6)	
Other	2 (1.6)	5 (2.9)	7 (2.3)	
Total	129 (100)	175 (100)	304 (100)	
Marital status				
Single	61 (47.3)	71 (40.6)	132 (43.4)	0.506^ǂ^
Married	66 (51.2)	101 (57.7)	167 (54.9)	
Divorced	2 (1.6)	3 (1.7)	5 (1.6)	
Total	129 (100)	175 (100)	304 (100)	
Nationality				
Non-Saudi	36 (27.9)	37 (21.1)	73 (24)	0.172^†^
Saudi	93 (72.1)	138 (78.9)	231 (76)	
Total	129 (100)	175 (100)	304 (100)	
Mother had profession, now or in the past; n (%)				
No	63 (48.8)	111 (63.8)	174 (57.4)	0.009*^†^
Yes	66 (51.2)	63 (36.2)	129 (42.6)	
Total	129 (100)	174 (100)	303 (100)	
Age in Years				
Single	28.4 ± 5.7	27 ± 2.6	36.3 ± 9.4	0.129^ɫ^
Married	34.9 ± 9.4	37.2 ± 9.3	27.6 ± 4.4	0.069^ɫ^
Divorced	27 ± 1.4	30.3 ± 2.3	29 ± 2.5	0.174^ɫ^
Total	31.7 ± 8.5	32.9 ± 8.8	32.4 ± 8.7	0.233^ɫ^

*P-value<0.05

ɫ Independent Sample T-test

†Chi-square test

ǂLikelihood-ratio Chi-square test

Of 304 participants, a significantly higher proportion of women had working mothers as compared to men (51.2% vs 36.2%, p-value = 0.009, Table 1). Also, younger respondents (≤30 years) had a higher proportion of working mothers than the older respondents (49.7% vs 32.5%, p-value = 0.003, Table A in S1 File).

Of 213 Saudi doctors, 73 (34.3%) had been sponsored by the government of Saudi Arabia for study or training to a western country with almost equal proportion between males and females (35.4% vs 32.5%, p-value = 0.669, Table B in S1 File). Nearly 92% of the Saudi consultants were trained in a western country through Saudi government sponsorship (Table B in S1 File).

### Average daily hours of work, housework and sleep

There were no differences in the self-reported daily working hours (8.5 ± 1.9) and sleeping hours per night (5.9 ± 1.1) amongst men and women. However, significant gender differences existed in self-reported daily household and caring for family hours. Women reported spending significantly more hours on housework and caring for family than their male counterparts (5.3±3 vs 4.2±2.6, p-value = 0.02, Table C in S1 File). Married women with children spent more hours on household work and caring for family as compared to their married female colleagues without children (5.9±3.2 vs 3.5±1.5, p-value = 0.004, Table C in S1 File) A significantly higher proportion of females spending ≥4 hours on housework and family were of the opinion that female doctors should have less duty hours than their male counterparts as compared to females spending <4 hours (35.1% vs 14.3%, p-value = 0.035 Table D in S1 File).

#### Gender equality and social support at the work place for doctors of similar nationality, qualifications, professional level, competence and years of experience

A higher proportion of male participants believed salaries, hospital benefits, and the opportunity to enter any field of Medicine, Pediatrics or Surgery are equivalent for both male and female doctors, though not statistically significant (Table 2).

**Table 2:** Perception of doctors regarding gender equality between doctors who are of similar nationality, qualifications, professional level, competence, and years of experience at King Khalid University Hospital.

	Gender		Total	P-value
	Female	Male		
	n (%)	n (%)	n (%)	
Salaries are equivalent				
Agree	90 (69.8)	134 (76.6)	224 (73.7)	0.412^†^
Neutral or Uncertain	23 (17.8)	24 (13.7)	47 (15.5)	
Disagree	16 (12.4)	17 (9.7)	33 (10.9)	
Total	129 (100)	175 (100)	304 (100)	
Hospital benefits are equivalent				
Agree	71 (55)	118 (67.4)	189 (62.2)	0.061^†^
Neutral or Uncertain	27 (20.9)	22 (12.6)	49 (16.1)	
Disagree	31 (24)	35 (20)	66 (21.7)	
Total	129 (100)	175 (100)	304 (100)	
Duty hours are equivalent				
Agree	103 (80.5)	122 (69.7)	225 (74.3)	0.101^†^
Neutral or Uncertain	10 (7.8)	19 (10.9)	29 (9.6)	
Disagree	15 (11.7)	34 (19.4)	49 (16.2)	
Total	128 (100)	175 (100)	303 (100)	
There is equal opportunity for entering any field of Medicine/Pediatrics				
Agree	82 (63.6)	126 (72)	208 (68.4)	0.207^†^
Neutral or Uncertain	27 (20.9)	24 (13.7)	51 (16.8)	
Disagree	20 (15.5)	25 (14.3)	45 (14.8)	
Total	129 (100)	175 (100)	304 (100)	
There is equal opportunity for entering any field of Surgery				
Agree	39 (30.5)	74 (42.3)	113 (37.3)	0.091^†^
Neutral or Uncertain	34 (26.6)	43 (24.6)	77 (25.4)	
Disagree	55 (43)	58 (33.1)	113 (37.3)	
Total	128 (100)	175 (100)	303 (100)	
Female doctors should have less duty hours than their male counterparts				
Agree	35 (27.1)	26 (14.9)	61 (20.1)	0.003*^†^
Neutral or Uncertain	16 (12.4)	12 (6.9)	28 (9.2)	
Disagree	78 (60.5)	137 (78.3)	215 (70.7)	
Total	129 (100)	175 (100)	304 (100)	
There is equal opportunity for promotion				
Agree	59 (45.7)	116 (66.3)	175 (57.6)	0.002*^†^
Neutral or Uncertain	35 (27.1)	29 (16.6)	64 (21.1)	
Disagree	35 (27.1)	30 (17.1)	65 (21.4)	
Total	129 (100)	175 (100)	304 (100)	
There is favoritism by male supervisors towards females in performance evaluations, grades and promotions				
Agree	16 (12.4)	101 (57.7)	117 (38.5)	0.000**^†^
Neutral or Uncertain	37 (28.7)	44 (25.1)	81 (26.6)	
Disagree	76 (58.9)	30 (17.1)	106 (34.9)	
Total	129 (100)	175 (100)	304 (100)	

*P-value<0.05

**P-value<0.0001

†Chi-square test

A higher proportion of male doctors agreed with the statement that promotion opportunities are the same for both male and female doctors (66.3% vs 45.7%, p-value = 0.002); and that there was favoritism by male supervisors towards female doctors in performance evaluations, grades and promotions (57.7% vs 12.4%, p-value<0.0001, Table 2).

A higher proportion of non-Saudis as compared with Saudis thought that salaries, hospital benefits and duty hours are not equivalent for both male and female doctors (26% vs 6.1%, p-value<0.0001; 24.7% vs 20.8%, p-value = 0.034 and 24.7% vs 13.5%, p-value = 0.007 respectively; Table E in S1 File).

Regardless of gender and nationality, more than half of the participants believed they receive informational and instrumental support from their department and supervisors. (55.6% and 53.9%, Table 3 and Table F in S1 File) respectively. However, mixed responses were obtained when asked about whether supervisors provide emotional support during times of stress or unhappiness (p-value = 0.582; Table 3) A significantly higher proportion of non-Saudi doctors as compared to Saudi doctors agreed that supervisors provided emotional support when needed (47.9% vs 35.5%; p-value = 0.041; Table F in S1 File).

**Table 3:** Perception amongst doctors regarding support provided to them by their department and supervisors at King Khalid University Hospital.

	Gender		Total	P-value
	Female	Male		
	n (%)	n (%)	n (%)	
Supervisors provide emotional support during times of stress or unhappiness				
Agree	52 (40.3)	65 (37.1)	117 (38.5)	0.582^†^
Neutral or Uncertain	16 (12.4)	29 (16.6)	45 (14.8)	
Disagree	61 (47.3)	81 (46.3)	142 (46.7)	
Total	129 (100)	175 (100)	304 (100)	
Department provides informational support such as departmental updates and guidance				
Agree	72 (55.8)	97 (55.4)	169 (55.6)	0.157^†^
Neutral or Uncertain	26 (20.2)	23 (13.1)	49 (16.1)	
Disagree	31 (24)	55 (31.4)	86 (28.3)	
Total	129 (100)	175 (100)	304 (100)	
Supervisors provide instrumental support and help in finishing a task, if need be				
Agree	65 (50.4)	99 (56.6)	164 (53.9)	0.422^†^
Neutral or Uncertain	25 (19.4)	25 (14.3)	50 (16.4)	
Disagree	39 (30.2)	51 (29.1)	90 (29.6)	
Total	129 (100)	175 (100)	304 (100)	

†Chi-square test

#### Female doctors working in a non-segregated environment

More than half of the female doctors (n = 67 (52.3%)) reported that they never wear the veil, 46 (35.9%) always wear the veil, whereas 15 (11.8%) wear the veil either inside or outside the hospital. Of those who wore the veil (n = 61), the majority (88.5%) were Saudis (p-value<0.0001).

Over half the women doctors stated that, under their white coats, they wore a skirt and blouse (54.7%); followed by scrubs (27.3%) (Table 4). A significantly higher proportion of female doctors than male agreed that female doctors could wear scrubs if they preferred (91.5% vs 77.7%; p-value = 0.006) and that the face veil prevents optimal doctor to patient communication (32.6% vs 20%; p-value = 0.008, Table 4). However, a higher proportion of male doctors felt that all female doctors should cover their face as they examine male patients (16.6% vs 6.2%; p-value<0.0001, Table 4).

**Table 4:** Hospital attire of women doctors at King Khalid University Hospital.

	Marital status			P-value
Regarding the face veil (niqab)	Married	Single/divorced	Total	
	n (%)	n (%)	n (%)	
I do not wear the veil	38 (58.5)	29 (46)	67 (52.3)	0.159^†^
I wear the veil	27 (41.5)	34 (54)	61 (47.7)	
Total	65 (100)	63 (100)	128 (100)	
Dress code	Nationality			P-value
	Non-Saudis	Saudis	Total	
Scrubs	7 (20)	28 (30.1)	35 (27.3)	0.013*^†^
Skirt & blouse	15 (42.9)	55 (59.1)	70 (54.7)	
Trousers & blouse	13 (37.1)	10 (10.8)	23 (18)	
Total	35 (100)	93 (100)	128 (100)	
	Gender		Total	P-value
	Female	Male		
Female trainees can wear scrubs under white coats				
Agree	118 (91.5)	136 (77.7)	254 (83.6)	0.006*^†^
Neutral or Uncertain	7 (5.4)	22 (12.6)	29 (9.5)	
Disagree	4 (3.1)	17 (9.7)	21 (6.9)	
Total	129 (100)	175 (100)	304 (100)	
Veil prevents optimal doctor to patient communication since the doctor's face is hidden				
Agree	42 (32.6)	35 (20)	77 (25.3)	0.008*^†^
Neutral or Uncertain	27 (20.9)	28 (16)	55 (18.1)	
Disagree	60 (46.5)	112 (64)	172 (56.6)	
Total	129 (100)	175 (100)	304 (100)	
All female doctors should wear the veil since they examine male patients				
Agree	8 (6.2)	29 (16.6)	37 (12.2)	0.000**^†^
Neutral or Uncertain	17 (13.2)	41 (23.4)	58 (19.1)	
Disagree	104 (80.6)	105 (60)	209 (68.8)	
Total	129 (100)	175 (100)	304 (100)	
Male and female doctors' attire should comply with infection control guidelines of keeping sleeves above the wrists and pinning head scarves and veils.				
Agree	85 (65.9)	123 (70.3)	208 (68.4)	0.530*^†^
Neutral or Uncertain	32 (24.8)	34 (19.4)	66 (21.7)	
Disagree	12 (9.3)	18 (10.3)	30 (9.9)	
Total	129 (100)	175 (100)	304 (100)	

*P-value<0.05

**P-value<0.0001

†Chi-square test

Regardless of gender, most of the participants were of the opinion that female doctors appear confident and male and female doctors interact comfortably in a mutually respectful environment, which is safe for female doctors. (Table 5).

**Table 5:** Aspects of working in a mixed environment for male and female doctors at King Khalid University Hospital.

	Gender			P-value
	Female	Male	Total	
	n (%)	n (%)	n (%)	
Female doctors appear to be as confident in their work environment as do the male doctors.				
Agree	105 (81.4)	125 (71.4)	230 (75.7)	0.128^†^
Neutral or Uncertain	10 (7.8)	23 (13.1)	33 (10.9)	
Disagree	14 (10.9)	27 (15.4)	41 (13.5)	
Total	129 (100)	175 (100)	304 (100)	
Male and female doctors appear to interact comfortably with each other in the work environment.				
Agree	99 (76.7)	138 (78.9)	237 (78)	0.908^†^
Neutral or Uncertain	13 (10.1)	16 (9.1)	29 (9.5)	
Disagree	17 (13.2)	21 (12)	38 (12.5)	
Total	129 (100)	175 (100)	304 (100)	
Male and female doctors appear to work in a mutually respectful environment.				
Agree	107 (82.9)	150 (85.7)	257 (84.5)	0.068^†^
Neutral or Uncertain	15 (11.6)	9 (5.1)	24 (7.9)	
Disagree	7 (5.4)	16 (9.1)	23 (7.6)	
Total	129 (100)	175 (100)	304 (100)	
Male doctors resent female doctors that are senior to them and do not like taking orders from them.				
Agree	22 (17.1)	23 (13.1)	45 (14.8)	0.007*^†^
Neutral or Uncertain	40 (31)	31 (17.7)	71 (23.4)	
Disagree	67 (51.9)	121 (69.1)	188 (61.8)	
Total	129 (100)	175 (100)	304 (100)	
Female doctors are safe and secure in the hospital environment during their night duties when on-call.				
Agree	90 (69.8)	134 (76.6)	224 (73.7)	0.223^†^
Neutral or Uncertain	20 (15.5)	26 (14.9)	46 (15.1)	
Disagree	19 (14.7)	15 (8.6)	34 (11.2)	
Total	129 (100)	175 (100)	304 (100)	
Female doctors can examine the male urogenital system.				
Agree	47 (36.4)	94 (53.7)	141 (46.4)	0.008*^†^
Neutral or Uncertain	40 (31)	34 (19.4)	74 (24.3)	
Disagree	42 (32.6)	47 (26.9)	89 (29.3)	
Total	129 (100)	175 (100)	304 (100)	
Male doctors can examine the female urogenital system.				
Agree	59 (45.7)	99 (56.6)	158 (52)	0.046*^†^
Neutral or Uncertain	36 (27.9)	29 (16.6)	65 (21.4)	
Disagree	34 (26.4)	47 (26.9)	81 (26.6)	
Total	129 (100)	175 (100)	304 (100)	

*P-value<0.05

†Chi-square test

A significantly higher proportion of male doctors were of the opinion that it is acceptable for female doctors to examine the male urogenital system and male doctors to examine the female urogenital system (53.7% vs 36.4%, p-value = 0.008 and 56.6% vs 45.7%, p-value = 0.046 respectively-Table 5). Overall, of 304 participants, 133 (43.8%) agreed that doctors can examine the urogenital system of the opposite sex.

Regarding whether female doctors face harassment from male doctors, a higher proportion of female doctors ≤30 years of age agreed with the statement as compared to female doctors >30 years (20.7% vs 6.4%, p-value = 0.036, Table 6) whereas, regardless of age, most of the male doctors disagreed with the statement (63.2%, p-value = 0.881, Table 6).

**Table 6:** Harassment of women doctors at King Khalid University Hospital.

Gender		Age		Total	P-value
		≤30	>30		
		n (%)	n (%)	n (%)	
Female	Female doctors face harassment by male doctors				
	Agree	17 (20.7)	3 (6.4)	20 (15.5)	0.036*^†^
	Neutral or Uncertain	26 (31.7)	12 (25.5)	38 (29.5)	
	Disagree	39 (47.6)	32 (68.1)	71 (55)	
	Total	82 (100)	47 (100)	129 (100)	
Male	Agree	14 (14.4)	10 (13)	24 (13.8)	0.881*^†^
	Neutral or Uncertain	21 (21.6)	19 (24.7)	40 (23)	
	Disagree	62 (63.9)	48 (62.3)	110 (63.2)	
	Total	97 (100)	77 (100)	174 (100)	
Overall	Agree	31 (17.3)	13 (10.5)	44 (14.5)	0.201^†^
	Neutral or Uncertain	47 (26.3)	31 (25)	78 (25.7)	
	Disagree	101 (56.4)	80 (64.5)	181 (59.7)	
	Total	179 (100)	124 (100)	303 (100)	
Female	Female doctors face harassment by male patients				
	Agree	37 (45.1)	10 (21.3)	47 (36.4)	0.002*^†^
	Neutral or Uncertain	28 (34.1)	14 (29.8)	42 (32.6)	
	Disagree	17 (20.7)	23 (48.9)	40 (31)	
	Total	82 (100)	47 (100)	129 (100)	
Male	Agree	32 (33)	14 (18.2)	46 (26.4)	0.061^†^
	Neutral or Uncertain	38 (39.2)	32 (41.6)	70 (40.2)	
	Disagree	27 (27.8)	31 (40.3)	58 (33.3)	
	Total	97 (100)	77 (100)	174 (100)	
Overall	Agree	69 (38.5)	24 (19.4)	93 (30.7)	0.000**^†^
	Neutral or Uncertain	66 (36.9)	46 (37.1)	112 (37)	
	Disagree	44 (24.6)	54 (43.5)	98 (32.3)	
	Total	179 (100)	124 (100)	303 (100)	

*P-value<0.05

**P-value<0.0001

†Chi-square test

When asked about whether female doctors face harassment from male patients, we found that regardless of gender, a significantly higher proportion of young doctors of age ≤30 agreed with the statement as compared to those >30 years of age (38.5% vs 19.4%, p-value<0.0001 Table 6)

#### Social aspects for female doctors

Almost half of both male and female doctors did not believe that women are perceived as more eligible for marriage by becoming doctors (49.7% vs 48.8%, p-value = 0.375). A significantly higher proportion of male doctors agreed that if female doctors marry someone with a medical background, it will be better for marital harmony (68% vs 45%, p-value<0.0001). A significantly higher proportion of male doctors were of the opinion that female doctors should contribute, alongside men, to the household and family expenses (72.6% vs 57.4%, p-value = 0.012, Table 7).

**Table 7:** Social Aspects for Women Doctors at King Khalid University Hospital n = 304.

	Gender		Total	P-value
	Female	Male		
	n (%)	n (%)	n (%)	
Women improve their eligibility for marriage by becoming doctors				
Agree	13 (10.1)	26 (14.9)	39 (12.8)	0.375^†^
Neutral or Uncertain	53 (41.1)	62 (35.4)	115 (37.8)	
Disagree	63 (48.8)	87 (49.7)	150 (49.3)	
Total	129 (100)	175 (100)	304 (100)	
If female doctors earn more than their husbands, it may impact negatively on the marriage due to resentment of the husband.				
Agree	45 (34.9)	60 (34.3)	105 (34.5)	0.677^†^
Neutral or Uncertain	44 (34.1)	53 (30.3)	97 (31.9)	
Disagree	40 (31)	62 (35.4)	102 (33.6)	
Total	129 (100)	175 (100)	304 (100)	
It would be better for female doctors to marry someone with a medical background for greater marital understanding and harmony.				
Agree	58 (45)	119 (68)	177 (58.2)	0.000**^†^
Neutral or Uncertain	25 (19.4)	32 (18.3)	57 (18.8)	
Disagree	46 (35.7)	24 (13.7)	70 (23)	
Total	129 (100)	175 (100)	304 (100)	
Female doctors should not work while their children are <6 years old				
Agree	31 (24)	46 (26.3)	77 (25.3)	0.634^†^
Neutral or Uncertain	29 (22.5)	45 (25.7)	74 (24.3)	
Disagree	69 (53.5)	84 (48)	153 (50.3)	
Total	129 (100)	175 (100)	304 (100)	
Female doctors should contribute to the household and family expenses				
Agree	74 (57.4)	127 (72.6)	201 (66.1)	0.012*^†^
Neutral or Uncertain	25 (19.4)	27 (15.4)	52 (17.1)	
Disagree	30 (23.3)	21 (12)	51 (16.8)	
Total	129 (100)	175 (100)	304 (100)	
Female doctors should have the permission to drive since they may be required to in a medical emergency				
Agree	105 (81.4)	115 (65.7)	220 (72.4)	0.006*^†^
Neutral or Uncertain	14 (10.9)	27 (15.4)	41 (13.5)	
Disagree	10 (7.8)	33 (18.9)	43 (14.1)	
Total	129 (100)	175 (100)	304 (100)	
Female doctors are as equally committed to their careers as are male doctors.				
Agree	119 (92.2)	118 (67.4)	237 (78)	0.000**^†^
Neutral or Uncertain	8 (6.2)	21 (12)	29 (9.5)	
Disagree	2 (1.6)	36 (20.6)	38 (12.5)	
Total	129 (100)	175 (100)	304 (100)	
Female doctors are more likely to leave their jobs than men				
Agree	42 (32.6)	99 (56.9)	141 (46.5)	0.000**^†^
Neutral or Uncertain	30 (23.3)	39 (22.4)	69 (22.8)	
Disagree	57 (44.2)	36 (20.7)	93 (30.7)	
Total	129 (100)	174 (100)	303 (100)	
If I were to choose all over again, I would still choose to become a doctor.				
Agree	91 (70.5)	119 (68)	210 (69.1)	0.790^†^
Neutral or Uncertain	13 (10.1)	22 (12.6)	35 (11.5)	
Disagree	25 (19.4)	34 (19.4)	59 (19.4)	
Total	129 (100)	175 (100)	304 (100)	

*P-value<0.05

**P-value<0.0001

†Chi-square test

Almost all of the female participants, compared to males, were of the opinion that female doctors are as committed to their careers as are males (92.2% vs 67.4%, p-value<0.0001, Table 7). A significantly higher proportion of male doctors were of the opinion that female doctors are more likely to leave their jobs (56.9% vs 32.6%, p-value<0.0001, Table 7). However, of 304, 53 (17.4%) had taken a gap in their professional working life of which 16 (30.2%) had taken a gap due to family obligations, other than maternity leave, with almost equal proportion between males and females (male: 7(30.4%) and female: 9(31%), p-value = 0.010; Table A in S1 File)

Of 304 participants, 210 (69.1%) said that if they were asked to choose again, they would still choose to become a doctor with almost equal proportions between males and females (68% vs 70.5%, p-value = 0.790).

In comparison to male doctors, a significantly higher proportion of female doctors agreed that female doctors should have the permission to drive (65.7% vs 81.4%, p-value = 0.006).

## Discussion

Our survey shows that regardless of gender or nationality, the majority of participants were of the opinion that there was gender equality amongst doctors in terms of salaries, benefits, duty hours, performance evaluations and opportunities for promotion.

There exist about 415 governmental and 127 private hospitals in Saudi Arabia. [32] The Saudi Ministry of Civil Affairs mandates a fixed pay scale according to the professional level, years of experience, and nationality for all government doctors, irrespective of gender. [33, 34] Our results for gender parity in pay could therefore be extrapolated to Government doctors throughout Saudi Arabia.

A recent study from the United States found that female physicians at 24 public medical schools earned an average of 8% less than males. [35] Similarly, a pay gap for women in Medicine exists in the United Kingdom. [27]

Though most of our respondents thought that there were equal opportunities for promotion, with over half of male doctors believing that females were actually favored, we found that only about one-third of the consultants were females. A 2004 study of Saudi women in academic medicine similarly found that promotion to higher ranks lagged behind males. This was attributed to family responsibilities, lack of institutional support, and bias. [36] Equally in the West, despite parity in numbers, a disproportionately lower number of women achieve leading medical positions. [14, 20, 23, 25, 26, 28, 29] One of the pre-requisites for a consultant position at King Saud University is training abroad, usually at a recognized western institution. [37] Sponsorship by the Kingdom of Saudi Arabia for study abroad covers travel, tuition, insurance and living expenses for recipients, their spouses and children. [38] In 2013, of 126,745 total Saudi students studying abroad, 21,599 were in the discipline of Health and Social Sciences, of which 35.2% were Saudi women. [39] We found that an approximately equal proportion of male and female doctors had received sponsorship at some stage of their training. Nevertheless, women doctors are likely to have greater family responsibilities by the time they are required to pursue further training abroad for consultant position and may not be in a position to avail the opportunity, though equally available to them by law. Furthermore, women may not have the support of their male guardians, despite the recent royal decree relaxing male guardianship laws, thus allowing women to benefit from government services such as education and healthcare without requiring permission. [40] The lower representation of women in leading positions could also be a reflection of the slower entry of women in smaller numbers into the medical field. However, if this deficiency is to be remedied, the rules of promotion need to be relaxed for deserving women doctors in view of family obligations and/or restrictions, provision of greater institutional support for women with children and improving and recognizing national training programs.

Only about one-third of our respondents felt that females have equal opportunity to enter any field of surgery. Indeed, there are few female surgeons in Saudi Arabia [19]. In the United States, women still represent only 36% of training and 15% of practicing surgeons. [41, 42] A study of 334 women surgeons in the USA revealed that the majority perceived gender-based discrimination during medical school, residency and practice. [43]

We found no difference in daily working hours, averaging 8.5, between male and female doctors. A survey in the United States of 1049 academic physicians found that women spent 8.5 more hours per week on domestic activities and were more likely to take time off for child care than men. [44] A 2016 survey in the United States found that women worked shorter hours than men. [45] A 2013 Canadian study reported that among women, but not men, marriage and raising children resulted in fewer work hours. [46] Moreover, a 2000 study reported that women were 1.6 times more likely to experience burnout than men, their risk increasing with additional hours above a 40 hour week. [47] In a study from Saudi Arabia, of 174 female doctors, 43.1% felt they had not achieved a satisfactory balance between career and family, and 51.7% felt that their work impacted negatively on their family relationships. [48] Despite these well recognized challenges for women, only a minority of our respondents advocated shorter working hours for female doctors or not working at all while they have small children. Apart from maternity leave, our study did not find that more females than males had undertaken a gap in their professional life due to family obligations. Our study also demonstrated that the majority of females are committed to their careers and confident of their choice. A USA 2016 survey noted that only 58% of women said they would choose medicine again, and 38% their own specialty. [45] In a major study in *JAMA*, women tended to be more satisfied than men with their specialty but less satisfied with their level of autonomy, pay and resources. [49]

Studies to determine whether females are socially integrated in the profession of medicine suggest that women typically receive more emotional support than men but less informational and instrumental support by their department, supervisors and co-workers. [50] However, the results of this survey did not reveal any gendered patterns of support amongst our participants but requires more in-depth study.

Saudi Arabia is a conservative society and this culture is challenged daily by the mixed work environment of modern day hospitals. Requests for all-female hospitals have periodically emerged but gone unheeded. [51, 52] There are no special provisions for female doctors stipulated by the government of Saudi Arabia other than the dress code. The Ministry of Health requires women doctors to wear a head scarf and a white coat over modest attire, with most in our study preferring skirt and blouse. Surprisingly, irrespective of gender, there was a high acceptance of scrubs for female doctors, with only a minority disapproving. Only about one-third of our female respondents always wore the veil. Regarding doctors' attire from the patients' perspective, a 2012 Saudi study found that of 399 male and female patients, most (73%) preferred that female doctors wear long skirts with only 5.5% preferring scrubs and 39.8% preferring that female doctors cover their face. [53] In another Saudi study, about one-third (31.3%) of patients preferred that female doctors cover their face. [54] It is of concern that almost one-third of the doctors were not agreeable to conforming their hospital attire to good and simple infection control practices such as keeping sleeves above the wrists and pinning head scarves (worn by men and women), and veils.

A directive from the Ministry of Health allows male doctors to examine female patients in the presence of a nurse. [55] There are no laws against female doctors examining males. Despite legal sanction, our study demonstrated that less than half of our participants approved of doctors examining the urogenital system of the opposite sex. A 2016 study from the USA found a gender divide in the surgical practice of urology in that female surgeons operated on a significantly higher percent of female patients (54.4% vs 32.5%, p <0.01) and performed significantly more female specific procedures than their male counterparts (18 vs 10 per year, p <0.001). [56]

A minority of our respondents felt that female doctors were subject to harassment by male doctors and patients. A 2013 study of residents at three hospitals in Saudi Arabia found that sexual harassment was reported in 19.3% and significantly more by females (P = .0061). [57] A 2000 survey in the USA of 3000 full-time faculty members found that about half of the female faculty had experienced sexual harassment. [21] More recently, in a 2016 study in the USA of 1,066 physician-scientists, 30% of women reported sexual harassment and 66% gender bias in professional advancement. [58]

The average age of marriage for females is 25 years in Saudi Arabia. [59] Our study suggests that women doctors may be marrying later. Since one-third of our doctors were married to doctors, this may be another trend in the medical field, whereby doctors are finding their own partners as opposed to entering traditionally arranged marriages, but it requires further studies. Our study did not find that women doctors in Saudi Arabia are considered more eligible for marriage, which is unlike the situation in Pakistan, where doctor brides signify status. [60]

Although two-thirds of our participants were either unsure or disagreed that husbands could become resentful if their wives earned more, in western countries where women are increasingly on track to earn more than their husbands, this gender dynamic remains unresolved. [61] Indeed, in Saudi Arabia, women own 40% of family run companies, with over 44 billion USD in cash, funds and real estate investments. [62] They are catered to by women-only banks. In accordance with Islamic jurisprudence, women are not obligated to share their wealth with their family or husband or to provide for the household. [63] Almost one-fourth of our female doctors preferred to exercise this option though significantly more of the male doctors believed that females should share the economic burden.

No study of the social issues of any sector of women in Saudi Arabia can be complete without mention of the ban on women driving, which remains a major impediment to the successful integration of women in the economic domain. [62, 64] However, as predicted, economic reforms have catalyzed socio-cultural change and, by royal decree, the ban on women driving will be lifted in June 2018. [65, 66] Already, in the realm of politics, history was made whereby in 2015, women won seats in Saudi Arabia’s municipal polls which were the first ever elections open to female voters and candidates. [67] Likewise, in sports, Saudi women athletes competed in the Olympics for the first time in 2012 and 2016. This was followed by a reversal of a long standing ban on sports for women and girls in public schools which will now be offering physical education programs. [68, 69] These changes are in keeping with the goals of Vision 2030, an ambitious road map to reduce Saudi Arabia’s dependence on oil, diversify its economy and develop sectors such as health, education, infrastructure, sports, recreation and tourism. [70]

Several limitations of our study should be mentioned. Our response rate is not high, as is true for most physician surveys. [71] Moreover, less than half of our respondents were women. Our data is subjective self-reports based on perceptions of individual doctors whereas actual numerical data for variables such as salaries, benefits, grades, promotions and gender distribution in various fields would be more compelling. Our survey may be subject to some bias whereby respondents who are aware of the negative international image and scrutiny of Saudi Arabia in regards to women, wish to portray a more positive picture. Survey analysts have also shown that those with more positive experiences are more likely to answer surveys. This data could further be weakened if doctors were insecure regarding the confidentiality and anonymity of a survey that broached sensitive issues in a conservative society. Contractual expatriates, in particular, may be less bold than nationals in expressing their opinions. Nevertheless, there is a paucity of data on gender issues of females in medicine or any other field in Saudi Arabia. Our study addresses that deficiency at a time when increasing numbers of well-educated and highly qualified women are entering the workforce in Saudi Arabia. We believe that our study also begins to challenge the prevailing stereotype internationally of women in Saudi Arabia, a crucial step in promoting greater understanding amongst people of different cultures.

### Conclusion

Our survey of male and female doctors at a Government University hospital in Riyadh, Saudi Arabia, revealed that the majority of participants were of the opinion that there was gender equality amongst doctors in terms of salaries, benefits, duty hours, performance evaluations, opportunities for promotion and entry into any field of medicine or pediatrics, but not surgery. The majority also perceived women doctors to be working confidently in a mutually respectful, professional and safe environment, and to be as committed to their careers as male doctors. Women appear to be satisfied with their current status and the choice of their profession. However, there were significantly fewer women at consultant positions, a deficiency which must be addressed.

## Supporting information

S1 File Table A. Personal Information regarding region of birth in Saudi Arabia, profession of spouse and mother and whether any gap in professional life.
Table B: Sponsorship for education/training abroad by Kingdom of Saudi Arabia for Saudi nationals.
Table C: Average hours spent by doctors daily at work, doing housework and sleeping.
Table D: Household hours of work.
Table E: Perception amongst Saudi and non-Saudi doctors regarding gender equality between doctors who are of similar nationality, qualifications, professional level, competence, and years of experience at King Khalid University Hospital.
Table F: Perception amongst Saudi and non-Saudi doctors regarding support by their department and supervisors at King Khalid university hospital.
(DOCX)
