# Supplementary material for: Perceptions of gender equality, work environment, support and social issues for women doctors at a university hospital in Riyadh, Kingdom of Saudi Arabia
Source: PLoS One. 2017 Oct 26;12(10):e0186896. doi: 10.1371/journal.pone.0186896 (PMC5658081; doi:10.1371/journal.pone.0186896)
Supplement: S1 File — Table A. Personal Information regarding region of birth in Saudi Arabia, profession of spouse and mother and whether any gap in professional life. Table B: Sponsorship for education/training abroad by Kingdom of Saudi Arabia for Saudi nationals. Table C: Average hours spent by doctors daily at work, doing housework and sleeping. Table D: Household hours of work. Table E: Perception amongst Saudi and non-Saudi doctors regarding gender equality between doctors who are of similar nationality, qualifications, professional level, competence, and years of experience at King Khalid University Hospital. Table F: Perception amongst Saudi and non-Saudi doctors regarding support by their department and supervisors at King Khalid university hospital. (DOCX) [file pone.0186896.s001.docx]

| **Table A.** **Spouse is a doctor; gap in professional life, working mother and region of birth in Saudi Arabia** | | | | |
| --- | --- | --- | --- | --- |
|  | **Gender** | | | |
|  | **Female** | **Male** | **Total** | **P-value** |
|  | **n (%)** | **n (%)** | **n (%)** |  |
| **Spouse is a doctor** | | | | |
| **Yes** | 32 (47.1%) | 32 (31.1%) | 64 (37.4%) | 0.003^*†^ |
| **No** | 36 (52.9%) | 71 (68.9%) | 107 (62.6%) |  |
| **Total** | 68 (100) | 103 (100) | 171 (100) |  |
| **Reasons for gap in professional life** | | | | |
| Family obligations | 9 (31) | 7 (30.4) | 16 (30.8) | 0.010^*ǂ^ |
| Your personal ill health | 1 (3.4) | 2 (8.7) | 3 (5.8) |  |
| Study reasons | 11 (37.9) | 14 (60.9) | 25 (48.1) |  |
| Maternity leave | 8 (27.6) | 0 (0.0) | 8 (15.4) |  |
| Total | 29 (100) | 23 (100) | 52 (100) |  |
|  | **Was your mother ever employed, now or in the past:** | | | |
|  | **No** | **Yes** | **Total** | **P-value** |
|  | **n (%)** | **n (%)** | **n (%)** |  |
| **Age groups** | | | | |
| **<=30** | 90 (50.3) | 89 (49.7) | 179 (100) | 0.003^*†^ |
| **>30** | 83 (67.5) | 40 (32.5) | 123 (100) |  |
| **Total** | 173 (57.3) | 129 (42.7) | 302 (100) |  |
| **Country of birth KSA** | | | | |
| **Central** | 81 (51.3) | 77 (48.7) | 158 (100) | 0.001^*†^ |
| **East/West** | 28 (54.9) | 23 (45.1) | 51 (100) |  |
| **North/South** | 30 (85.7) | 5 (14.3) | 35 (100) |  |
| **Total** | 139 (57) | 105 (43) | 244 (100) |  |
| *P-value<0.05,†Chi-square test, ǂLikelihood-ratio Chi-square test | | | | |

| **Table B: Sponsorship for education/training abroad by Kingdom of Saudi Arabia for Saudi nationals only** | | | | |
| --- | --- | --- | --- | --- |
|  | **Have you ever been sponsored for study/training to a western country by the government of Saudi Arabia:** | | **Total** | **P-value** |
|  | **No** | **Yes** |  |  |
|  | **n (%)** | **n (%)** | **Count** |  |
| **Gender** | | | | |
| Female | 56 (67.5) | 27 (32.5) | 83 (100) | 0.669^†^ |
| Male | 84 (64.6) | 46 (35.4) | 130 (100) |  |
| Total | 140 (65.7) | 73 (34.3) | 213 (100) |  |
| **Position at KKUH** | | | | |
| Intern | 32 (84.2) | 6 (15.8) | 38 (100) | 0.000^**ǂ^ |
| Resident | 83 (77.6) | 24 (22.4) | 107 (100) |  |
| Fellow | 10 (100) | 0 (0) | 10 (100) |  |
| Consultant | 3 (8.1) | 34 (91.9) | 37 (100) |  |
| Senior Registrar/Associate Consultant | 5 (83.3) | 1 (16.7) | 6 (100) |  |
| Demonstrator | 4 (36.4) | 7 (63.6) | 11 (100) |  |
| Other | 3 (75) | 1 (25) | 4 (100) |  |
| Total | 140 (65.7) | 73 (34.3) | 213 (100) |  |
| *P-value<0.05,**P-value<0.0001,†Chi-square test, ǂLikelihood-ratio Chi-square test | | | | |

| **Table C. Average daily working hours, daily household hours and sleeping hours per night for doctors** | | | | | | | |
| --- | --- | --- | --- | --- | --- | --- | --- |
|  |  | **Average number of working hours per day** | | **Average number of hours spend daily on housework, caring for children and family member/s:** | | **Hours of sleep / night** | |
| **Marital status** | **Gender** | **Mean ±SD** | **P-value** | **Mean ±SD** | **P-value** | **Mean ±SD** | **P-value** |
| **Ever married** | **Female** | 8.3±1.9 | 0.39 | 5.3±3 | 0.020^*^ | 5.8±1.2 | 0.24 |
|  | **Male** | 8.5±2.1 |  | 4.2±2.6 |  | 6±1 |  |
| **Single** | **Female** | 8.4±2.1 | 0.46 | 3.7±2.3 | 0.32 | 6±1.3 | 0.25 |
|  | **Male** | 8.6±1.6 |  | 3.3±2.2 |  | 5.8±1.2 |  |
| **Gender** | **Have children** | **Mean ±SD** | **P-value** | **Mean ±SD** | **P-value** | **Mean ±SD** | **P-value** |
| **Female** | **Yes** | 8.2±2.0 | 0.58 | 5.9±3.2 | 0.004^*^ | 5.8±1.3 | 0.5 |
|  | **No** | 8.5±1.5 |  | 3.5±1.5 |  | 6±0.7 |  |
| **Male** | **Yes** | 8.5±2.2 | 0.49 | 4.4±2.7 | 0.3 | 6.1±1.0 | 0.371 |
|  | **No** | 8.8±1.7 |  | 3.7±2.2 |  | 5.8±1.0 |  |
| *P-value<0.05, Independent sample T-test | | | | | | | |

| **Table D. Household hours of work** | | | | |
| --- | --- | --- | --- | --- |
|  |  | | | **P-value** |
|  | **<4 hours** | **≥4 hours** | **Total** |  |
|  | **n (%)** | **n (%)** | **n (%)** |  |
| **Female doctors should have less duty hours than their male counterparts** | | | | |
| Agree | 7 (14.3) | 27 (35.1) | 34 (27) | 0.035^*†^ |
| Neutral/uncertain | 7 (14.3) | 7 (9.1) | 14 (11.1) |  |
| Disagree | 35 (71.4) | 43 (55.8) | 78 (61.9) |  |
| Total | 49 (100) | 77 (100) | 126 (100) |  |
| *P-value<0.05,†Chi-square test | | | | |

| **Table E: Perception amongst Saudi and Non-Saudi doctors regarding gender equality between doctors who are of similar nationality, qualifications, professional level, competence, and years of experience at King Khalid University Hospital** | | | | |
| --- | --- | --- | --- | --- |
|  | **Nationality** | | **Total** | **P-value** |
|  | **Non-Saudi** | **Saudi** |  |  |
|  | **n (%)** | **n (%)** | **n (%)** |  |
| **Salaries are equivalent** | | | | |
| Agree | 33 (45.2) | 191 (82.7) | 224 (73.7) | 0.000^**†^ |
| Neutral or Uncertain | 21 (28.8) | 26 (11.3) | 47 (15.5) |  |
| Disagree | 19 (26) | 14 (6.1) | 33 (10.9) |  |
| Total | 73 (100) | 231 (100) | 304 (100) |  |
| **Hospital benefits are equivalent** | | | | |
| Agree | 37 (50.7) | 152 (65.8) | 189 (62.2) | 0.034^*†^ |
| Neutral or Uncertain | 18 (24.7) | 31 (13.4) | 49 (16.1) |  |
| Disagree | 18 (24.7) | 48 (20.8) | 66 (21.7) |  |
| Total | 73 (100) | 231 (100) | 304 (100) |  |
| **Duty hours are same** | | | | |
| Agree | 44 (60.3) | 181 (78.7) | 225 (74.3) | 0.007^*†^ |
| Neutral or Uncertain | 11 (15.1) | 18 (7.8) | 29 (9.6) |  |
| Disagree | 18 (24.7) | 31 (13.5) | 49 (16.2) |  |
| Total | 73 (100) | 230 (100) | 303 (100) |  |
| **There is favoritism by male supervisors towards female doctors in evaluations.** | | | | |
| Agree | 45 (61.6) | 106 (46.1) | 151 (49.8) | 0.030^*†^ |
| Neutral or Uncertain | 14 (19.2) | 44 (19.1) | 58 (19.1) |  |
| Disagree | 14 (19.2) | 80 (34.8) | 94 (31) |  |
| Total | 73 (100) | 230 (100) | 303 (100) |  |
| **There is equal opportunity for entering any field of Medicine/Pediatrics** | | | | |
| Agree | 55 (75.3) | 153 (66.2) | 208 (68.4) | 0.272^†^ |
| Neutral or Uncertain | 11 (15.1) | 40 (17.3) | 51 (16.8) |  |
| Disagree | 7 (9.6) | 38 (16.5) | 45 (14.8) |  |
| Total | 73 (100) | 231 (100) | 304 (100) |  |
| **There is equal opportunity for entering any field of Surgery** | | | | |
| Agree | 31 (42.5) | 82 (35.7) | 113 (37.3) | 0.340^†^ |
| Neutral or Uncertain | 20 (27.4) | 57 (24.8) | 77 (25.4) |  |
| Disagree | 22 (30.1) | 91 (39.6) | 113 (37.3) |  |
| Total | 73 (100) | 230 (100) | 303 (100) |  |
| **Female doctors should have less duty hours** | | | | |
| Agree | 21 (28.8) | 40 (17.3) | 61 (20.1) | 0.104^†^ |
| Neutral or Uncertain | 6 (8.2) | 22 (9.5) | 28 (9.2) |  |
| Disagree | 46 (63) | 169 (73.2) | 215 (70.7) |  |
| Total | 73 (100) | 231 (100) | 304 (100) |  |
| **There is equal opportunity for promotion** | | | | |
| Agree | 40 (54.8) | 135 (58.4) | 175 (57.6) | 0.478^†^ |
| Neutral or Uncertain | 19 (26) | 45 (19.5) | 64 (21.1) |  |
| Disagree | 14 (19.2) | 51 (22.1) | 65 (21.4) |  |
| Total | 73 (100) | 231 (100) | 304 (100) |  |
| **There is favoritism by male supervisors towards females in performance evaluations, promotions, grades** | | | | |
| Agree | 8 (57.1) | 57 (71.3) | 65 (69.1) | 0.322^ǂ^ |
| Neutral or Uncertain | 3 (21.4) | 6 (7.5) | 9 (9.6) |  |
| Disagree | 3 (21.4) | 17 (21.3) | 20 (21.3) |  |
| Total | 14 (100) | 80 (100) | 94 (100) |  |
| *P-value<0.05,†Chi-square test | | | | |

| **Table F. Perception amongst Saudi and Non-Saudi doctors regarding support by their department and supervisors at King Khalid university hospital** | | | | |
| --- | --- | --- | --- | --- |
|  | **Nationality** | | **Total** | **P-value** |
|  | **Non-Saudi** | **Saudi** |  |  |
|  | **n (%)** | **n (%)** | **n (%)** |  |
| **Supervisors provide emotional support during times of stress or unhappiness** | | | | |
| Agree | 35 (47.9) | 82 (35.5) | 117 (38.5) | 0.041^*†^ |
| Neutral or Uncertain | 5 (6.8) | 40 (17.3) | 45 (14.8) |  |
| Disagree | 33 (45.2) | 109 (47.2) | 142 (46.7) |  |
| Total | 73 (100) | 231 (100) | 304 (100) |  |
| **Department provides informational support and guidance** | | | | |
| Agree | 48 (65.8) | 121 (52.4) | 169 (55.6) | 0.121^†^ |
| Neutral or Uncertain | 8 (11) | 41 (17.7) | 49 (16.1) |  |
| Disagree | 17 (23.3) | 69 (29.9) | 86 (28.3) |  |
| Total | 73 (100) | 231 (100) | 304 (100) |  |
| **Supervisors provide instrumental support and help in finishing a task, if needed.** | | | | |
| Agree | 44 (60.3) | 120 (51.9) | 164 (53.9) | 0.287^†^ |
| Neutral or Uncertain | 8 (11) | 42 (18.2) | 50 (16.4) |  |
| Disagree | 21 (28.8) | 69 (29.9) | 90 (29.6) |  |
| Total | 73 (100) | 231 (100) | 304 (100) |  |
| *P-value<0.05,†Chi-square test | | | | |
